# Effect of TENS on pain in relation to central sensitization in patients with osteoarthritis of the knee: study protocol of a randomized controlled trial

**DOI:** 10.1186/1745-6215-13-21

**Published:** 2012-02-21

**Authors:** David Beckwée, Willem De Hertogh, Pierre Lievens, Ivan Bautmans, Peter Vaes

**Affiliations:** 1 Vrije Universiteit Brussel, Department of Physiotherapy, Faculty of Physical Education & Physiotherapy, Laarbeeklaan 103, B-1090 Brussels, Belgium; 2 Artesis University College Antwerpen, Health Care Department, Rehabilitation Sciences and Physiotherapy; 3 Vrije Universiteit Brussel, Frailty in Ageing Research Department, Laarbeeklaan 103, B-1090 Brussels, Belgium

## Abstract

**Background:**

Central sensitization has recently been documented in patients with knee osteoarthritis (OAk). So far, the presence of central sensitization has not been considered as a confounding factor in studies assessing the pain inhibitory effect of tens on osteoarthritis of the knee. The purpose of this study is to explore the pain inhibitory effect of burst tens in OAk patients and to explore the prognostic value of central sensitization on the pain inhibitory effect of tens in OAk patients.

**Methods:**

Patients with knee pain due to OAk will be recruited through advertisements in local media. Temporal summation, before and after a heterotopic noxious conditioning stimulation, will be measured. In addition, pain on a numeric rating score, WOMAC subscores for pain and function and global perceived effect will be assessed. Patients will be randomly allocated to one of two treatment groups (tens, sham tens). Follow-up measurements will be scheduled after a period of 6 and 12 weeks.

**Discussion:**

Tens influences pain through the electrical stimulation of low-threshold A-beta cutaneous fibers. The responsiveness of central pain-signaling neurons of centrally sensitized OAk patients may be augmented to the input of these electrical stimuli. This would encompass an adverse therapy effect of tens. To increase treatment effectiveness it might be interesting to identify a subgroup of symptomatic OAk patients, i.e., non-sensitized patients, who are likely to benefit from burst tens.

**Trial Registration:**

ClinicalTrials.gov: NCT01390285

## Background

Osteoarthritis (OA) is characterized by damaged articular cartilage of synovial joints. About 17% of people aged over 45 years suffer from pain and loss of function due to symptomatic knee osteoarthritis (OAk) [1] and 40% of people aged over 65 years have symptomatic OA of the knee or hip [2,3]. The prevalence of arthritis and more especially OA increases with age [4]. Therefore, the direct health care costs associated with this disease will become a major burden in the near future as the proportion of elderly people in the population increases [5]. Because there is no cure for OA, the treatment is focused on reducing physical disability and impairment and controlling pain while minimizing the potentially harmful side effects of medications [6].

### The use of transcutaneous electrical nerve stimulation (tens) in the management of OAk patients

Transcutaneous electrical nerve stimulation (tens) is a non-pharmacological, inexpensive and safe form of analgesia [7]. The pain modulating effect of tens is assigned to peripheral components which may be regulated by central mechanisms [8]. The inhibitory effect of tens is based on the 'Gate Control Theory' of pain perception as described by Melzack and Wall [9]. This theory suggests that stimulation of large (A-beta) afferent cutaneous fibers activate the inhibitory-interneurons in the dorsal horn of de medulla. This may weaken the transmission of nociceptive signals from small diameter A-delta and C-fibers. As OA is a dynamic process that involves phases of inflammation with possible increase of pain during these phases, tens may then be indicated as a facilitator for exercise. The use of tens to relieve knee pain in osteoarthritis of the knee (OAk) is recommended in various clinical guidelines as a conservative treatment to relieve knee pain [10-12]. However, Rutjes et al. [13] conclude in their meta-analysis that adequate evidence to support the use of any type of transcutaneous electrostimulation in patients with knee osteoarthritis is lacking.

### Central sensitization in patients with osteoarthritis of the knee

Central sensitization is defined as "an augmentation of responsiveness of central pain-signaling neurons to input from low-threshold mechanoreceptors" [14]. Central sensitization embodies modified sensory processing in the brain and malfunctioning of descending pain-inhibitory mechanisms [15]. The importance of central sensitization as a potential underlying mechanism of pain in OAk has recently gained interest [16]. In the past decade suggestions were already made concerning the influence of sensitization of wide dynamic range (WDR) dorsal horn neurons when explaining an increase of the mechanical pain threshold after applying a blockage of A -beta nerve fibers [17]. Continuous and intense nociceptive input from the OA-damaged knee joint may encourage central sensitization [18,19] and is assumed to play an important role in OA [20]. Central sensitization in patients with OAk was detected by Arendt-Nielsen and colleagues [16] by enhanced temporal summation (TS) of pain and impaired diffuse noxious inhibitory control (DNIC). TS is defined as an increase in pain rating after repetitive stimulation at a constant stimulus intensity [21]. It is thought to be a psychophysiological correlate of wind-up, which is defined as the increase in response magnitude of second-order nociceptive neurons and higher structures [22] to repetitive noxious stimulation [23]. DNIC is a phenomenon in which pain from one part of the body inhibits pain elsewhere in the body [21]. It appears that multireceptive WDR neurons in the dorsal horn of the spinal cord and trigeminal nociceptive play a key role in DNIC inhibitory processes [24]. These neurons are important convergence sites for both excitatory and inhibitory influences arising from more than one type of tissue and can be activated by both innocuous and noxious stimuli [25]. It has been suggested that DNIC functions as a filter, which allows the system to focus on a painful stimulus in a background of basic somesthetic activity. The mechanism appears to involve the spinal cord as well as supraspinal regions [25].

### Sensitization in relation to the effect of tens

The effectiveness of tens on knee pain in OAk patients is unclear, due to conflicting results [13]. The ambiguous results of the included studies could be due to the inclusion of heterogeneous types of patients. One important feature distinguishing patient groups might be the presence of central sensitization.

In case of central sensitization, the response to peripheral stimuli, e.g., electrical stimuli as well as mechanical pressure, cold, heat, light, sound and chemical substances is enhanced [26]. Tens (Burst) currents influence pain through the electrical stimulation of low-threshold A-beta cutaneous fibers. This stimulation might enhance the responsiveness of central pain-signaling neurons of OAk patients who are centrally sensitized. In these patients an adverse effect of tens on the pain perception can be expected. The identification of potential subgroups of sensitized and non-sensitized symptomatic OAk patients is needed, as they can react differently to tens. So far, the presence of central sensitization has not been considered as a confounding factor in studies assessing the pain inhibitory effect of burst tens on OAk.

### Aim of the proposed study

This study has a double aim. First to explore the pain inhibitory effect of burst tens in OAk patients. Second, to assess whether central sensitization affects this pain inhibitory effect. A 6 week burst tens intervention period will be succeeded by a 12 week follow up period. As an international consensus definition for central sensitization is lacking [26], we aim to assess the central sensitization by measuring pressure pain thresholds (PPT) at the knee (local pain) and at the upper limb (spreading pain) [27] and temporal summation before and during a heterotopic noxious conditioning stimulation [16,21]. The test-retest reliability of TS and DNIC has been reported as acceptable [21].

## Methods

### Study design

A randomized controlled clinical trial with blinded assessment and a follow-up period of 12 weeks is developed. This study will be conducted at the university hospital of the Vrije Universiteit Brussel (UZ Brussel), Brussels, Belgium. The research protocol is approved by the Medical Ethics Committee of the university hospital of the Vrije Universiteit Brussel (UZ Brussel). Written and signed informed consent will be obtained from all participants.

### Study population

A community sample of OAk patients with knee pain will be recruited.

### In- and exclusion criteria

To be included, patients need to be over 50 years old. All should have osteoarthritis in at least one knee fulfilling the American College of Rheumatology classification criteria [28] and report peak knee pain of more than 3 on a Numeric Rating Score (0-10 scale) over the last 24 hours.

Patients are excluded if they have had a knee surgery or intra-articular corticosteroid or hyaluronic acid injection [29] within 6 months, current or past (within 4 weeks) oral corticosteroid use, a history of knee joint replacement or tibial osteotomy, in case of contraindications to burst tens (pacemakers, epilepsy, dermatological conditions, abnormal sensation in the knees, pregnancy) or if they are unable to apply tens independently [7].

### Recruitment

Patients will be recruited through advertisements placed in local media. All patients will initially be screened over the phone with regard to selection criteria. If appropriate they will undergo medical screening with a project rheumatologist.

Consequently, an appointment for the baseline measurements will be made. Due to ethical considerations, analgesia and non-steroidal anti-inflammatory drugs will be permitted (and registered) as required and as participants are used taking during the last month. All participants will be asked to refrain from seeking other forms of treatment during the trial. They will be questioned about having received other forms of treatment in the final stage of the protocol.

### Randomization and blinding

Following baseline measurements, subjects will be randomly allocated to one of two treatment groups, ie., tens or sham tens. However, all patients will be told that two different kinds of tens are being tested. Randomization will be performed in blocks of four, stratified by sex and age. The allocation of this will be done by an independent researcher who will not interfere with other experimental procedures. We will use two boxes: one for each sex. In each box, subgroups will be made for five age categories: 50-59; 60-69; 70-79; 80-89; 90-99 years. Four numbered cards will be put in each age category. The numbering of the cards starts at one and ends at 40 and each number will correspond to a tens or a sham tens treatment. At the start of the study, each age category contains two tens and two sham tens treatments. Each time a new patient is included, a card will be taken out of the corresponding box and age category. When the four cards of one category are used, all of them will be put back in the box, so that a second round can be started. At each round, the numbered cards will be differently assigned to a tens/sham tens treatment by an independent researcher. The measurements will be carried out by a different researcher than the one that informs the patients concerning the treatment modalities. The information of the baseline measurements will remain concealed until the end of the study ensuring blinded assessment.

### Sample size

Sample size calculation is based on data from Law and Cheing [30]. They found a significant difference in the reduction of pain (2.9(4.7) points on a 0-10 VAS) when applying a tens current (pulse width: 200 μs, frequency: 100 Hz) on the knee of patients with OAk compared to a control group not receiving tens treatment. In this study the response within each subject group was normally distributed with standard deviation 4.7. If the true difference in the experimental and control means is 2.9, we will need to study 42 experimental subjects and 42 control subjects to be able to reject the null hypothesis that the population means of the experimental and control groups are equal with probability (power) 0.8. The Type I error probability associated with this test of this null hypothesis is 0.05. To take into account a 15% loss of data sample size is inflated up to 50 per group (n/0.85). Sample size is augmented by 10% per potential confounding factor (i.e. age and sex) [31]. The total sample size to be recruited will be 120 subjects.

### Baseline measurements

Age, gender, BMI, duration of knee OA symptoms, medication use, previous treatment and surgery for knee OA will be obtained at baseline.

#### Knee pain and physical function

Overall average knee pain (KPa) and peak pain intensity over the last 24 h (KPp) will be assessed by a 11-point numeric rating scale with terminal descriptors of 0 = no pain; 10 = maximal pain [32]. Self-reported knee pain and difficulty with physical function will be measured using the Western Ontario and McMaster Universities (WOMAC) Index [32].

#### Central sensitization

Temporal summation as well as diffuse noxious inhibitory control will be used to detect central sensitization. The assessment will be done in accordance with the protocol of Cathcart et al. [21]. The outcome assessor will be trained by an experienced researcher prior to the start of the study. The intra-observer reliability of the outcome assessor will be tested in a subgroup of the participants.

#### Pressure pain threshold (PPT)

Pressure stimuli will be measured using a hand held digital algometer (Somedic AB, Farsta, Sweden). Pain detection thresholds will be measured on the knee (M. Vastus Medialis) and on the upper part of the homolateral arm (lateral part of M. Deltoideus, 10 cm below acromion) [33]. The pressure will be increased at a rate of approximately 1 kg/s. Subjects have to report when the feeling of pressure alone changes into a feeling of pressure and pain (Pain Detection Threshold). The mean of two measurements, taken 30 s apart from each other, will be used for further use (temporal summation).

#### Temporal summation (TS)

Temporal summation assessment will start 2 min after PPT measurements to avoid contamination by possible sensitization from the pain threshold stimulation. TS will be induced at the knee and arm through 10 pressure pulses with the hand held algometer (Somedic AB, Farsta, Sweden) at PPT intensity. For each pulse, pressure will be increased at a rate of 2 kg/s and held during 1 s. An interstimulus interval of 1 s will be applied. Patients will be instructed to rate their pain level according to a NRS at the first, 5th and 10th pulse [21].

#### Diffuse noxious inhibition control (DNIC)

Ischemic compression of the heterolateral arm will be used as heterotopic noxious conditioning stimulation to evoke diffuse noxious inhibition control (DNIC). This method has previously been described [16,21]. A tourniquet cuff will be applied on the upper heterolateral arm and inflated until a painful intensity. After an adaptation period of 30 s, patients will be asked to rate the pain on a NRS. Cuff inflation will then be increased or decreased until a pain intensity of 3 of 10 on the NRS is reached. The arm is then rested while TS assessment is repeated as described above.

### Interventions

The subjects that enter the tens -group will be asked to apply the tens therapy for at least 40 minutes continuously per day during 6 weeks. All patients in the intervention group will be informed that the intensity should be high enough so that an unpleasant but non-painfull sensation is acquired. The settings of the current will not change during the study. The patients that are assigned to the sham tens treatment will receive an inactive placebo tens therapy using a nonfunctional unit that appears to work but provides no stimulus. To blind the investigator as well as the patient, the sham tens device will deliver current for 30 seconds and then ramps down to no current. This approach has been studied and has been proven effective in blinding subjects and investigators to eliminate expectation bias [34]. All participants will be told that they may not feel anything after a while but that this does not mean that the machine is not working. All patients will be asked to come back after two and four weeks to assess their ability to precisely replicate the tens installation.

### Equipment

#### Composition and dosing

The settings of the current: burst tens with pulse width of 250 μsec; internal frequency: 100 Hz; burst frequency 3 Hz; intensity: until an unpleasant but non-painful sensation is acquired. The tens devices (ELPHA II 3000, FH Service) to be used will be pre-set with the current settings. Subjects will be taught how to switch on and off the device and how to augment the intensity (mA) of the burst tens current. Patients will be asked not to change the current settings. The electrodes will be placed in the dermatome L4 at 10 cm above the knee. Potential adverse effects may be irritation of the skin near the electrodes.

### Outcome assessment

#### Primary outcome variable

The change from baseline to final assessment of the study knee in the continuous variables *"overall average knee pain" (KPa) and "peak pain over the last 24 h" (KPp*) will be measured using a numeric rating scale [32].

#### Secondary outcome variables

The change from baseline to final assessment of the study knee in *self-reported knee pain and difficulty with physical function *will be measured using the WOMAC Index [32].

The *global perceived effect *(GPE) compared to baseline will be measured on an ordinal scale (1 - much worse, 2 - slightly worse, 3 - no change, 4 - slightly better, 5 - much better) [35]. The GPE is an overall measurement of the perceived effect of the patient. It has been used in various studies to assess therapeutic effects [36-38].

The change from baseline to final assessment of *medication use *will be recorded as another pain variable.

*Pressure pain threshold, temporal summation (TS) and *Diffuse noxious inhibition control (DNIC) will be used as baseline variables.

### Follow up

During the 6 weeks following the first appointment, a study nurse will contact the participants of the tens and stens groups weekly by telephone. Subjects will be asked about any inconvenience with the handling of the tens device as well as if they have received other treatments.

After 6 and 12 weeks participants will be asked to rate their GPE, as well as KPa, KPp, WOMAC and medication use. At these occasions *Pressure pain threshold, temporal summation (TS) and *Diffuse noxious inhibition control (DNIC) will be assessed as well.

### Data reduction and Statistical analysis

An intention-to-treat analysis will be performed so that the integrity of the randomization is ensured. Normality will be checked via the Shapiro-Wilk test.

To ensure good balance of participant characteristics in each group at baseline, stratification is used [39]. Changes in differences between both treatment groups after the intervention period (at 6 w and 12 w) will be analyzed using repeated measures ANOVA for the following variables: KPp, KPa, WOMAC subscores for pain and function, GPE. If the modulus of the partial correlation of potential confounding factors age and sex with the primary outcome values equals minimum 0.3, these covariates of prognostic values will be added as covariates in the ANOVA model [40].

As a secondary analysis, a linear regression analysis will be used with the following dependent factors: BMI, TS and DNIC; and as independent factors: KPa, KPp, WOMAC subscales for pain and function.

Significance value for all tests will be set at p < 0.05. All analyses will be performed using SPSS 20 for Windows.

## Discussion

This study has a double aim. First, to explore the pain inhibitory effect of tens in OAk patients. Second, to assess whether components of central sensitization like temporal summation and diffuse noxious inhibitory control, affect this pain inhibitory effect.

It is believed that tens influences pain through different pathways. One of these pathways is the gate-control theory [9]. The second goal of our study protocol, i.e. exploring the potential prognostic value of TS and DNIC on the pain inhibitory effect of tens, is based on this rationale. However, opioid pathways that involve peripheral, spinal and supraspinal mechanisms [41,42] are also proposed as an explanation for the pain modulation of tens and this pathway may be less vulnerable for an adverse effect of tens in central sensitized OAk patients.

As tens may influence pain through the electrical stimulation of low-threshold A-beta cutaneous fibers, the responsiveness of central pain-signaling neurons of OAk patients who are centrally sensitized is augmented to the input of these electrical stimuli. This would encompass an adverse therapy effect of tens on the pain perception in patients with OAk who are centrally sensitized. Therefore we think that it might be interesting to identify a subgroup of symptomatic OAk patients, i.e., non-sensitized patients, who are likely to benefit from burst tens.

The majority of studies that were published in the past and that focused on treatment effects of tens, embody currents that were administered by a therapist in a practice or hospital setting. As portable tens devices are marketed as small, inexpensive, easy-to-use home units [13], we choose to use a self-administered protocol. This approach may encompass a positive influence on the patient's participation to the treatment as well as to the cost effectiveness of the treatment, as the machines that are used in (professional) practices are far more expensive than these portable tens devices.

To assess whether dose-effects may influence the treatment outcome, we plan to record the daily duration of the electro stimulation that is applied.

One major concern when using self-administered medical care is treatment adherence. We think that a weekly phone call after the initial start of the study may improve treatment adherence as well as stimulate participants to consistently fill out the diary.

We have chosen a daily treatment duration of 40 minutes continuously. This is based on the findings of Cheing and colleagues [43]. They found that the cumulative analgesic effect manifested by a tens group that received 40 minutes of tens therapy was significantly greater than those seen in 2 other active tens groups (20 and 60 minutes application) in the follow-up session, i.e. 2 weeks after termination of the 2 week treatment. They conclude that 40 minutes is the optimal treatment duration of tens to be used for the relief of pain in patients with knee osteoarthritis.

The results of this study will not only provide insight into the effect of tens but they may contribute to future studies investigating the identification of patient subgroups that may benefit from tens.

## List of abbreviations used

BMI: Body Mass Index; DNIC: Diffuse Noxious Inhibitory Control; GPE: Global Perceived Effect; KPa: overall average knee pain intensity; KPp: peak knee pain intensity; NRS: Numeric Rating Scale; OA: Osteoarthritis; Oak: Osteoarthritis of the knee; PPT: Pressure Pain Threshold; Tens: Transcutaneous Electrical Nerve Stimulation; TS:Temporal Summation; Stens: sham Transcutaneous Electrical Nerve Stimulation; WOMAC: Western Ontario and McMaster Universities; OI: Osteoarthritis Index; WDR: Wide Dynamic Range.

## Competing interests

The authors declare that they have no competing interests.

## Authors' contributions

DB drafted the manuscript.

WDH has been involved in drafting the manuscript, revised it critically for important intellectual content and made substantial contributions to conception and design of the manuscript.

PL revised the manuscript critically for important intellectual content.

IB made substantial contributions to conception and design of the manuscript and revised it critically for important intellectual content.

PV has been involved in drafting the manuscript and revised it critically for important intellectual content.

All authors have given final approval of the version published.
